# The transcriptome of mouse central nervous system myelin

**DOI:** 10.1038/srep25828

**Published:** 2016-05-13

**Authors:** Sudhir Thakurela, Angela Garding, Ramona B. Jung, Christina Müller, Sandra Goebbels, Robin White, Hauke B. Werner, Vijay K. Tiwari

**Affiliations:** 1 Institute of Molecular Biology (IMB), 55128 Mainz, Germany; 2 Department of Neurogenetics, Max Planck Institute of Experimental Medicine, 37075 Göttingen, Germany; 3 Institute of Physiology, University Medical Center of the Johannes Gutenberg-University, 55128 Mainz, Germany

## Abstract

Rapid nerve conduction in the CNS is facilitated by insulation of axons with myelin, a specialized oligodendroglial compartment distant from the cell body. Myelin is turned over and adapted throughout life; however, the molecular and cellular basis of myelin dynamics remains elusive. Here we performed a comprehensive transcriptome analysis (RNA-seq) of myelin biochemically purified from mouse brains at various ages and find a surprisingly large pool of transcripts enriched in myelin. Further computational analysis showed that the myelin transcriptome is closely related to the myelin proteome but clearly distinct from the transcriptomes of oligodendrocytes and brain tissues, suggesting a highly selective incorporation of mRNAs into the myelin compartment. The mRNA-pool in myelin displays maturation-dependent dynamic changes of composition, abundance, and functional associations; however ageing-dependent changes after 6 months were minor. We suggest that this transcript pool enables myelin turnover and the local adaptation of individual pre-existing myelin sheaths.

The biogenesis of myelin has long been viewed as a purely developmental process. Indeed, myelin is one of the most long-lived structures of the rodent brain^1^. However, the finding that several myelin proteins display a half-life of about 6 months indicates that myelin is indeed turned over in normal brains, though slowly. Utilizing the fallout of nuclear bomb tests in the 1950s and 1960s as a global labeling pulse, the normal turnover of oligodendrocytes and myelin has also been assessed in the human brain by quantifying the levels of the carbon isotope ^14^C in autopsy material from deceased subjects^2^. In the analyzed white matter tract (the corpus callosum), a continuous but very slow turnover of oligodendrocytes was observed. Indeed, nearly all white matter oligodendrocytes are born in the first five years of human life and afterward turned over remarkably slowly. However, the turnover rate of myelin was considerably higher than what would be predicted if entirely owing to the replacement of old myelin sheaths by adult-born oligodendrocytes. Together, this has suggested that existing oligodendrocytes remodel their myelin over time. Compared to the corpus callosum, the turnover of oligodendrocytes is higher in the grey matter of the human brain^2^, suggestive of region-dependent myelin changes that may also account for the formation of new myelin sheaths by adult-born oligodendrocytes in the rodent optic nerve^3^. Additional to what is required for normal myelin turnover, adult myelination by existing mature oligodendrocytes can be triggered by cellular stimuli that induce a net growth of pre-existing myelin sheaths^4^,^5^.

Myelin growth occurs at ‘myelin sheath assembly sites’ (MSAS)^6^, necessitating the presence or biogenesis of future myelin constituents in the non-compact compartments of myelin, which are connected to their distant oligodendroglial cell bodies by tenuous cellular processes. Indeed, two major routes of future constituents into myelin have been identified. First, future myelin membrane can be transported in vesicles^7^, which is slow owing to the long distance from the oligodendrocytic cell body to the myelin sheath and further limited by the closure of myelinic channels through compact CNS myelin coinciding with its maturation^8^. Secondly, myelin constituents can be synthesized by local translation, i.e. at MSAS in non-compact myelin. This was shown for Myelin Basic Protein (MBP)^9^, an abundant structural myelin protein^10^ that is rate-limiting for CNS myelination^11^,^12^. By associating with and thereby neutralizing membrane phospholipids^13^,^14^,^15^, MBP allows the close approximation of adjacent myelin membrane surfaces^16^. Indeed, oligodendrocytes lacking MBP fail in the formation of compact CNS myelin, e.g., in *Mbp*^*shiverer*^ mice^17^,^18^. The trafficking of *Mbp*-mRNA along microtubules into oligodendroglial processes^19^,^20^ and the inhibition of its premature translation are tightly controlled by a multitude of factors contained in transport granules^21^,^22^,^23^. Consequently, *Mbp*-mRNA has been found at MSAS^24^ and enriched in biochemically purified myelin compared to brain lysates, along with transcripts encoding the unrelated myelin-oligodendrocyte basic protein (MOBP) and ferritin heavy chain (FTH1)^6^.

Considering that the turnover and adaptation of myelin require the availability and selective incorporation of many more proteins into the future myelin sheath we sought to systematically determine by RNA-Sequencing (RNA-Seq) the mRNAs present in CNS myelin. Surprisingly, we find that adult CNS myelin comprises an unexpectedly large number of mRNAs. Developmentally, the myelin transcriptome reaches maturity by six months of age. According to functional genomic analysis, myelin is enriched for mRNAs encoding myelin proteins, components of the translational machinery, and molecules required for protein transport and localization.

## Results

### Purified myelin is enriched for myelin markers both at the protein and transcript level

**Figure 1:**
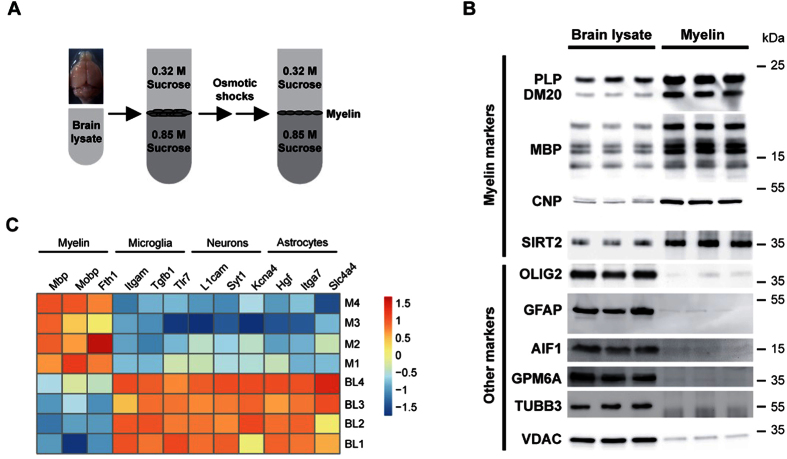
Myelin proteins and mRNAs are enriched in purified myelin compared to brain homogenate. (**A**) Scheme illustrating the biochemical purification of a light-weight membrane fraction enriched for myelin by homogenizing mouse brains in 0.32 M sucrose, sequential sucrose density gradient centrifugation, and osmotic shocks. Myelin accumulates at the interface between 0.32 M and 0.85 M sucrose. (**B**) Immunoblot analysis of myelin-enriched fractions and equal amounts of brain lysate to compare the abundance of marker proteins for compact myelin (PLP/DM20, MBP), non-compact myelin (CNP, SIRT2), the oligodendroglial nuclear/cytoplasmic compartment (OLIG2), astrocytes (GFAP), microglia (AIF1/IBA1), neuronal plasma membrane (GPM6A), axonal microtubules (TUBB3/TUJ1), and mitochondria (VDAC). Blot represents three biological replicates (male c57Bl6/N mice, age P75). Note that myelin markers were enriched in purified myelin while markers of other cellular sources were reduced. (**C**) Heatmap displaying reverse CT values from qRT-PCRs for three markers each specific for myelin, migroglia, neurons and astrocytes performed on myelin biochemically purified from the brains of 4 individual mice (M1–4) compared to the respective brain lysates (BL1–4) at six month of age.

We have biochemically enriched myelin from mouse brains by sucrose density gradient centrifugation (Fig. 1A), reproducibly yielding a myelin fraction enriched for classical marker proteins of both the compact (PLP, MBP) and the non-compact compartments of myelin (CNP, SIRT2) (Fig. 1B)^25^,^26^,^27^. In contrast, marker proteins for the oligodendroglial nuclear/cytoplasmic compartment (OLIG2), astrocytes (GFAP), microglia (AIF1), neurons (GPM6A, TUBB3), and mitochondria (VDAC) were reduced in purified myelin compared to brain lysates (Fig. 1B). When subjecting purified myelin to RNA isolation, reverse transcription, and qRT-PCR, the abundance of the myelin-related mRNAs *Mbp, Mopb,* and *Fth1* was strongly increased compared to brain lysates (Fig. 1C), in agreement with a previous report using Northern blots^6^. Importantly, transcripts specific to neurons (*L1cam, Syt1, Kcn4*), astrocytes (*Hgf, Itga7, Slc4a4*) or microglia (*Itgam, Tgfb1, Tlr7*) were diminished in myelin compared to brain lysate according to qRT-PCR analysis (Fig. 1C). Together, both myelin proteins and myelin-related transcripts are strongly enriched in biochemically purified myelin.

### The myelin transcriptome displays a distinct composition

**Figure 2:**
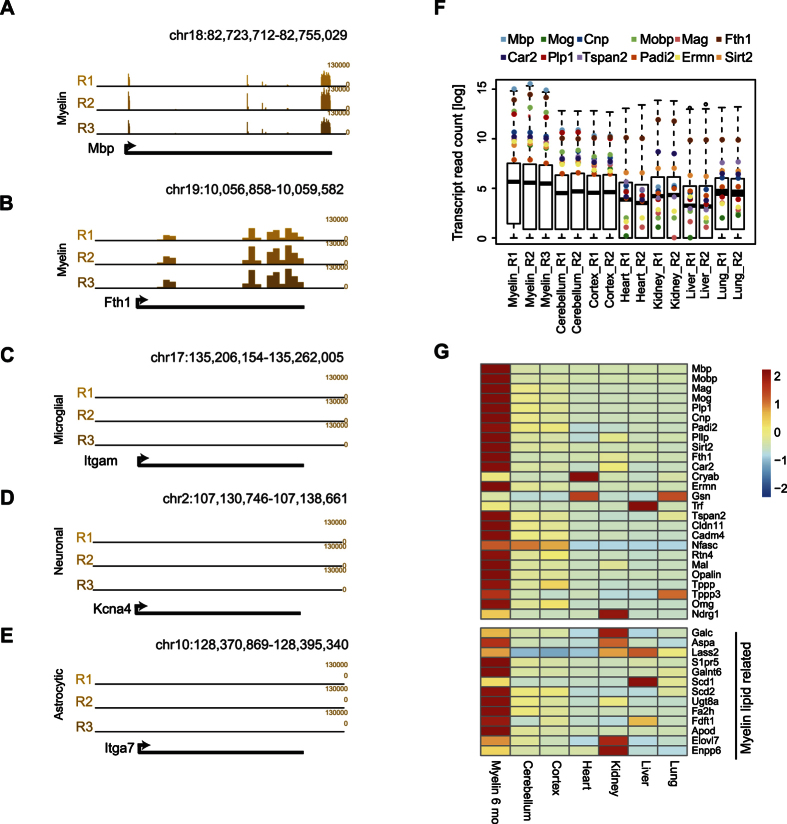
Transcripts encoding known myelin proteins are enriched in mature CNS myelin. (**A–E**) UCSC browser tracks of selected RNA-seq mRNA-reads on purified myelin. Displayed are two myelin-related genes (*Mbp, Fth1*), one microglial marker (*Itgam*), one neuronal marker (*Kcn4a*) and one astrocytic marker (*Itga7*). Note that mature myelin-related mRNAs were detected. Also see Fig S1C-K. (**F**) Box-Whisker plots showing transcript read count of all mRNAs identified in myelin purified from male c57Bl6/N mice (age 6 months) compared to total cerebellum, cortex, heart, kidney, liver, and lung. The three biological replicates display similar transcript distribution (R1-R3) and selected myelin-related mRNAs (highlighted) are enriched in myelin compared to the other samples. (**G**) Heat map showing the abundance of selected myelin-related transcripts in myelin (age 6 months), cerebellum, cortex, heart, kidney, liver, and lung. Note that mRNAs encoding enzymes related to myelin lipids are also enriched in myelin.

To determine the composition of the entire mRNA-pool present in CNS myelin, we performed RNA-Seq on myelin extracted from three individual mouse brains (male c57Bl6/N, age 6 months) (Supplementary Table 1). We observed a very strong replicate correlation (Fig. S1A,B). The reads reproducibly mapped to the exons of selected myelin-related genes (Fig. 2A,B and Fig. S1 D–O), indicating that indeed mature transcripts were detected. However, the abundance of transcripts ageencoding for microglial, neuronal and astrocytic marker genes was very low (Fig. 2C–E), implying the detection of myelin-specific transcripts with low contamination by transcripts originating from neighboring cells.

**Figure 3:**
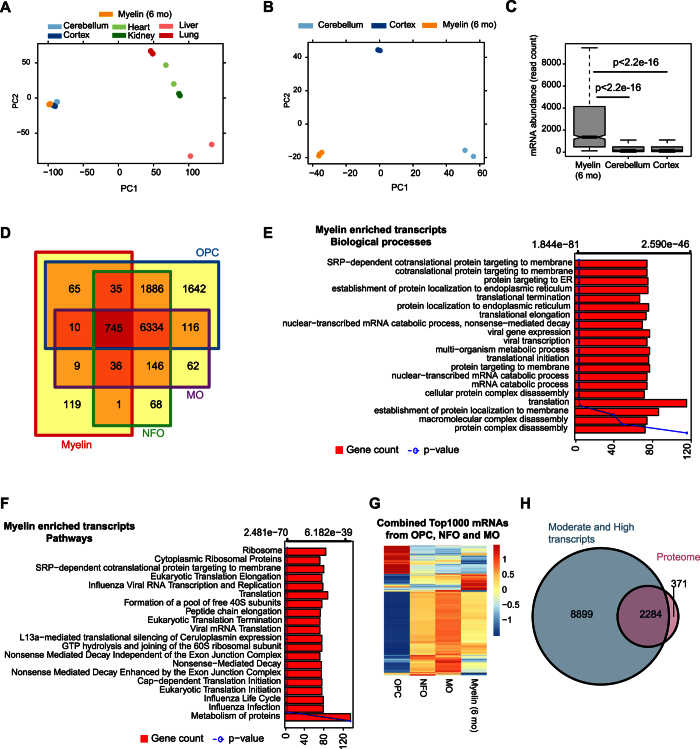
The transcriptome of mature CNS myelin. (**A**) Principal component analysis (PCA) comparing the transcriptome of myelin with that of total cerebellum, cortex, heart, kidney, liver, and lung. First two principal components (PC) are represented as distances on x- and y-axis to display variation between samples. Myelin and brain samples cluster away from those of other germ layers. (**B**) PCA comparing the myelin transcriptome with cerebellum and cortex. Note the similarity of the three biological replicates of myelin. (**C**) Box-Whisker plots displaying the relative abundance of mRNAs that are at least 2-fold enriched in myelin compared to cerebellum and cortex (n = 1020). (**D**) Comparison of mRNAs that are at least 2-fold enriched in myelin compared to cerebellum and cortex with the mRNAs expressed in oligodendrocyte progenitor cells (OPC), newly formed oligodendrocytes (NFO), and myelinating oligodendrocytes (MO) according to Zhang *et al*. 2014. Only a subset of the mRNAs expressed in the oligodendrocyte lineage is enriched in myelin. (**E**) Bar plot showing biological processes associated with myelin-enriched transcripts. Bars indicate the number of genes in a category (x-axis); the line gives the p-value for each biological process. (**F**) Bar plot showing biological pathways associated with myelin-enriched transcripts. Bars indicate the number of genes in a category (x-axis); the line gives the p-value for each pathway. (**G**) Heat map of the top 1000 most abundant mRNAs expressed in the oligodendrocyte lineage (combined OPC, NFO, and MO) to compare their abundance with that in myelin. mRNAs expressed in the oligodendrocyte lineage are not necessarily enriched in myelin, and myelin-enriched mRNAs are not necessarily among the most abundantly expressed oligodendroglial mRNAs. (**H**) Venn diagram comparing the mass spectrometrically identified proteins in purified myelin with the mRNAs detected in myelin at a high or moderate level at the same age (6 months).

The transcript pool of myelin is overall more similar to that of total brain regions (cerebellum, cortex) compared to tissues derived from other germ layers (Fig. 2F,G and Fig. S2 and Fig. S3A and Fig. 3A). Importantly, in depth analysis confirmed the strong enrichment in myelin of mRNAs (*Mbp, Mobp, Fth1*) previously analyzed in a small-scale study^6^. For a surprising number of known myelin proteins, the abundance of the corresponding mRNA in purified myelin was considerably higher compared to cerebellum and cortex homogenates, as well as to non-neuronal tissues (Fig. 2F,G). Also many transcripts encoding myelin lipid-related enzymes were enriched in myelin (Fig. 2G). Interestingly, the transcripts most abundant in myelin (Fig. S2) comprise several mRNAs for which to the best of our knowledge no relation to central myelination has yet been reported, including *Plekhb1, Bcas1, Trp53inp2*, and *Ptgds*. Together, myelin displays a specific transcript composition and abundance profile clearly distinct from other transcriptomes (Fig. 2F,G and Fig S2 and Fig. 3A,B).

To further elucidate the composition of the myelin transcriptome we focused on myelin-enriched transcripts, i.e. those mRNAs with a normalized read count of >100 and that were at least 2-fold more abundant in myelin compared to total cerebellum (1141 transcripts), cortex (846), or both (1020) (Fig. S3B) (Supplementary Table 2, 3). Many of these transcripts were enriched in myelin to an unexpectedly high degree (Fig. 3C and Fig. S3C,D).

Next we compared the myelin transcriptome with that of the myelinating cells of the CNS. Here, we took advantage of recently published genome-wide expression data^28^ for oligodendrocyte progenitor cells (OPC), newly formed oligodendrocytes (NFO), and myelinating oligodendrocytes (MO) purified by immunopanning. Density plots of these datasets revealed overall similarly distributed transcript abundances, allowing to categorize mRNAs as not expressed, lowly expressed, moderately expressed, or highly expressed (Fig. S3E). When comparing all moderately and highly abundant transcripts (i.e., with an FPKM >2 at any stage of the oligodendrocyte lineage; n = 11155) with mRNAs enriched in myelin compared to both cerebellum and cortex (n = 1020) we found that most myelin-enriched transcripts are expressed at all stages of oligodendroglial maturation (n = 745) (Fig. 3D). A minority of myelin-enriched mRNAs was found expressed only after the OPC stage (n = 46); this category includes many of the classical myelin markers. The myelin-enriched mRNAs (n = 1020) largely represent biological processes and pathways strongly associated with protein translation and targeting (Fig. 3E,F), suggesting that the myelin sheath comprises transcripts required for its own translation machinery.

Notably, a very large number of mRNAs expressed in oligodendrocytes are not enriched in myelin (n = 10254) (Fig. 3D). We thus compared in a heat map the top 1000 most abundant oligodendroglial mRNAs with their abundance in myelin (Fig. 3G and Fig. S3F) (Supplementary Table 4). As expected, the mRNA-abundance profiles of NFO and MO were more similar to each other than to OPC. Strikingly, mRNAs highly abundant in NFO and MO were not necessarily among the most abundant mRNAs in myelin, and *vice versa* mRNAs highly abundant in myelin were not necessarily among the most abundant oligodendroglial mRNAs (Fig. 3G and Fig S3F). For example, when comparing the mRNAs highly expressed in myelinating oligodendrocytes (FPKM > 64) (according to the dataset by Zhang and colleagues^28^) with those that are of low abundance or below threshold in myelin (Supplementary Table 5), the strongest depletion was found for *BC002163, Gm6682, Fam57a*, and *Gm6788*. On the other hand, the most abundant mRNAs in myelin (*Mbp, Fth1, Plekhb1*, and *Mobp*) were all enriched in myelin compared to myelinating oligodendrocytes. Together, oligodendroglial cells and the myelin compartment display clearly distinct mRNA-profiles, suggesting that the mechanisms to transport mRNAs into the myelin sheath are selective. Importantly, the most abundant myelin mRNAs largely clustered away from the mRNAs most abundant in neurons, astrocytes and microglia (according to the dataset by Zhang and colleagues) (Fig. S3G), thereby supporting qRT-PCR data gained for individual marker genes (Fig. 1C) and thus the notion that myelin displays a distinct transcriptome.

To systematically assess whether mRNAs present in myelin can be ultimately translated into myelin proteins, we mass spectrometrically analyzed myelin purified from the brains of 6 months old mice (male, c57Bl/6N) in which 2655 distinct proteins were identified (Supplementary Table 6). For the vast majority of them (n = 2284; 86%), the corresponding mRNA was detected in myelin at a moderate or high level (Fig. 3H) (Supplementary Table 7). Among those were a considerable number of metabolic proteins largely associated with the nucleotide metabolism, as well as proteins associated with biological processes like membrane organization, protein localization and transport (Fig. S3H). When comparing the myelin proteome with the entire myelin transcriptome including mRNAs of low abundance, 88% (n = 2327) of the identified myelin proteins were also detected at the level of the corresponding mRNA (Fig. S3I). Together this indicates that the transcriptome and the proteome of CNS myelin correspond with each other surprisingly well.

### Developmental maturation of the myelin transcriptome

**Figure 4:**
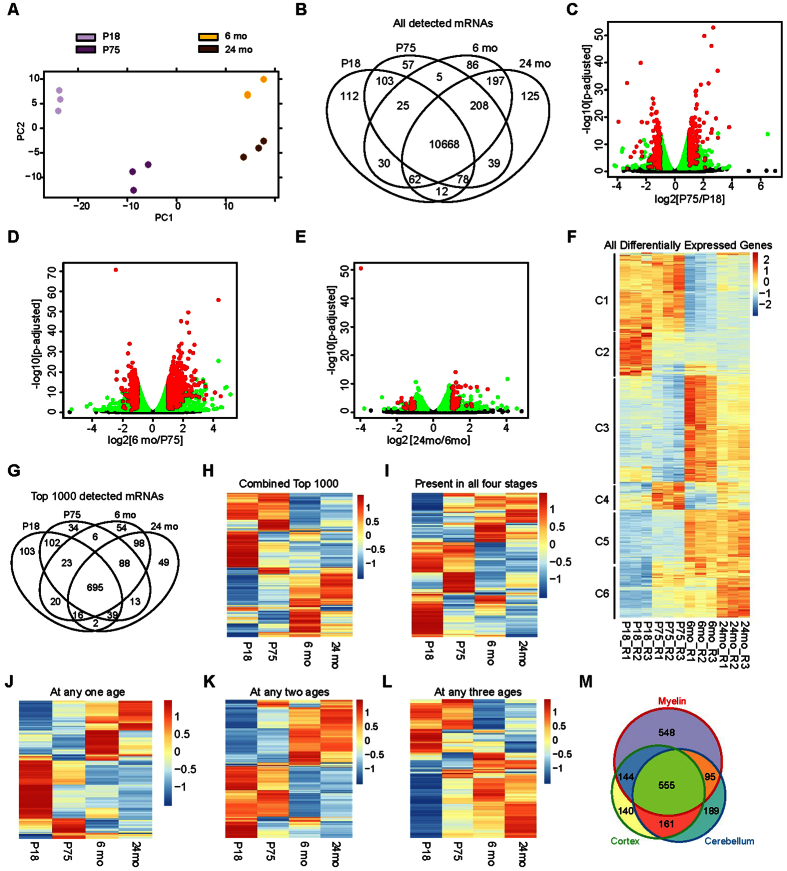
Developmental maturation of the myelin transcriptome. (**A**) PCA plot showing transcriptome dynamics of myelin at four different ages (postnatal day 18 (P18), P75, 6 months and 24 months). (**B**) Venn diagram comparing all myelin mRNAs at specified ages (normalized read count; threshold >100 reads). (**C**) Volcano plot displaying transcripts with abundance change in myelin >2-fold between P18 and P75. X-axis displays log2-fold change between P18 and P75, and y-axis shows respective adjusted p-value as -log10. Red dots: transcripts that display fold-change abundance of at least 2-fold (203 increasing; 158 decreasing), green dots: other significantly changing transcripts, black dots: transcripts without significant change in abundance. (**D**) Same as in Fig. 4C showing mRNAs of which the abundance in myelin changes over 2-fold between P75 and 6 months of age. (**E**) Same as Fig. 4C showing mRNAs of which the abundance in myelin changes over 2-fold between 6 and 24 months of age. (**F**) Heat map of all mRNAs of which the abundance in myelin changes over 2-fold in any of the analyzed subsequent ages. (**G**) Venn diagram comparing the transcripts in myelin that are among the top 1000 most abundant at any of the analyzed ages (n = 1342) revealing 695 mRNAs among the top 1000 at all ages. (**H**) Heat map showing relative abundance of the combined top 1000 most abundant transcripts in myelin at any analyzed age (n = 1342 as in Fig. 3G). (**I**) Heat map of transcripts that were among the top 1000 most abundant mRNAs in myelin at all four analyzed ages (n = 695, subset of Fig. 3H, as in Fig. 3G). (**J**) Heat map of transcripts that were among the top 1000 mRNAs in myelin at only one of the analyzed ages (n = 240, subset of Fig. 3H). (**K**) Heat map of transcripts among the top 1000 at any two ages (n = 241, subset of Fig. 3H). (**L**) Heat map of transcripts among the top 1000 at any three ages (n = 166, subset of Fig. 3H). (**M**) Venn diagram comparing combined top 1000 myelin transcripts at any age (n = 1342 as in Fig. 3G) with the top 1000 transcripts cortex and cerebellum.

We next asked whether the myelin transcriptome is fully matured already in newly formed myelin or if it undergoes developmental changes. Therefore we determined the myelin transcriptome of mouse brains at different ages (P18, P75, 6 and 24 months). Interestingly, the myelin transcriptome was very similar when comparing the latter ages while we observed clear developmental differences between P18, P75, and 6 months of age (Fig. 4A). This suggests that the myelin transcriptome in mice reaches a mature state by about 6 months of age.

To elucidate the transcript profile in myelin irrespective of age-dependent changes we compared all transcripts (normalized read count >100) of each analyzed age. As expected, a large number of transcripts (n = 10668) was present at all analyzed ages (Fig. 4B). The top 1000 most abundant among these transcripts were preferentially associated with biological processes as protein translation, transport, and localization (Fig. S4A), biological pathways as ribosome biogenesis and assembly (Fig. S4B), and known mouse mutant phenotypes as abnormal nervous system physiology and morphology (Fig. S4C).

To further explore developmental changes affecting the mRNA-abundance profile of myelin in the context of the observation that the myelin transcriptome reaches maturity at 6 months of age we performed more detailed differential analysis of the consecutive ages. Indeed, considerable changes of the myelin transcriptome were observed until 6 months of age (Fig. 4C,D), whereas changes were comparatively minor between 6 and 24 months of age (Fig. 4E) (Supplementary Table 8–10).

By gene ontology (GO) term enrichment analysis, the mRNAs differentially present in the myelin transcriptome at P18 and P75 are associated with protein targeting and localization, translational termination, and cell adhesion (Fig. S4D). The transcripts that distinguish myelin at P75 and at 6 months of age are preferentially associated with ion transport, cell-signaling, protein transport and localization (Fig. S4E). Importantly, although the myelin transcriptome was largely similar at 6 and 24 months of age (Fig. 4E), a class of transcripts that distinguished both ages was associated with immune response (Fig. S4F).

**Figure 5:**
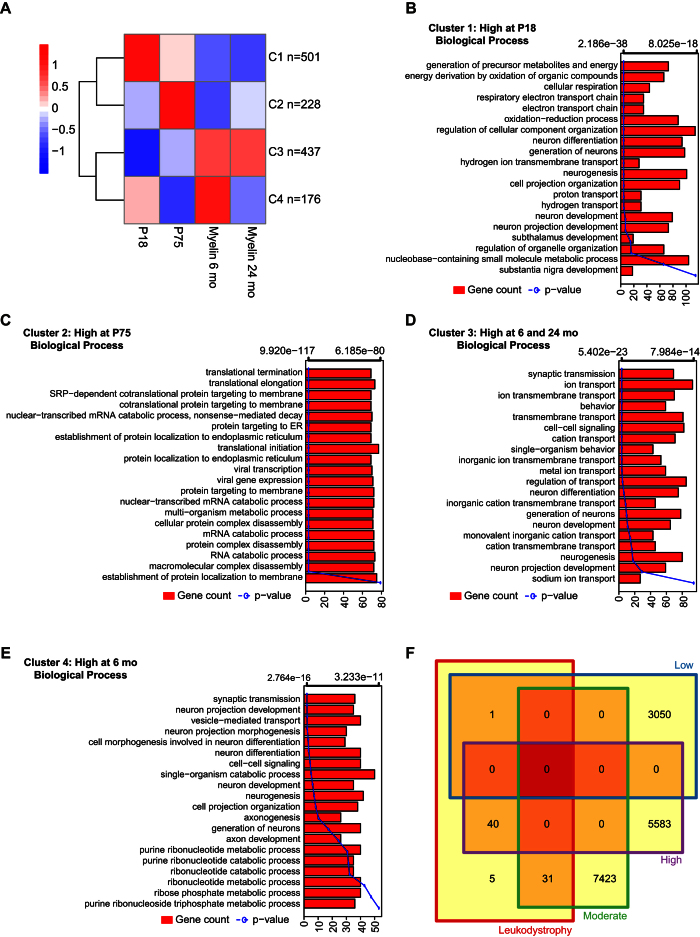
Functional genomics of the myelin transcriptome. (**A**) Heat map showing distinct k-means clusters of transcripts in myelin of which the abundance differentially changes between any analyzed age. (**B**) Bar plot showing enrichment of biological processes for mRNAs in cluster 1 obtained from k-means clustering (compare Fig. S3G) representing abundant transcripts in myelin at age P18. Bars indicate numbers of mRNAs in the respective biological process (lower x-axis) while the line represents the respective p-value (upper x-axis). (**C**) Same as in Fig. 5B but for k-means clustered transcripts abundant in myelin at age P75. (**D**) Same as in Fig. 5B but for k-means clustered transcripts abundant in myelin at 6 and 24 months of age. (**E**) Same as in Fig. 5B but for k-means clustered transcripts abundant in myelin at 6 months of age. (**F**) Comparison of the mRNAs detected in myelin with the genes causing (when mutated) heritable disorders preferentially affecting the white matter of the brain (leukodystrophies/leukoencephalopathies). Note that surprisingly many leukodystrophy-associated mRNAs were identified in myelin at least at moderate or high abundance (see also supplemental Fig. S5).

To better resolve the maturation of the myelin transcriptome we visualized in a heat map all mRNAs that displayed significant abundance changes in any pairwise comparison (Fig. 4F). Hierarchical clustering resulted in six clusters of mRNAs based on their developmental abundance kinetics (Fig. 4F). We observed transcripts that are only highly abundant in newly formed myelin (P18: cluster 2, C2) or myelin at both P18 and P75 (C1). The cluster of transcripts most abundant at only P75 (C4) was comparatively small. A consistent increase in mRNA abundance was seen for another comparatively small cluster (C6). The largest clusters consisted of mRNAs with the highest abundance at 6 or 24 months with only moderate changes between these ages (C3, C5) (Fig. 4F). To further elucidate changes of the myelin transcriptome between the four analyzed ages we also performed k-means clustering (Fig. 5A), which yielded defined clusters for myelin at P18 (C1), P75 (C2), and 6 months (C4), as well as a cluster at both 6 and 24 months of age (C3) (Supplementary Table 11). Interestingly, no distinct cluster was gained for myelin at 24 months of age, supporting the observation that the myelin transcriptome has largely reached maturity by 6 months of age.

To further explore the transcripts highly abundant in myelin (Supplementary Table 12) at particular or all analyzed ages (compare Fig. 4G), we plotted only these mRNAs and observed dynamically changing transcript levels coinciding with myelin maturation but comparatively moderate changes between 6 and 24 months of age (Fig. 4H,I). Among the analyzed ages, myelin at P18 comprises the largest group of uniquely abundant transcripts, which appear specific to newly formed myelin (Fig. 4J). A small cluster comprises transcripts specifically abundant in myelin at P75 (Fig. 4J). The transcripts constituting the larger clusters in myelin at 6 or 24 months are commonly also present at high abundance in the respective other later age but not in myelin at P18 or P75 (Fig. 4J and Fig. S4G), again supporting that the myelin transcriptome matures by 6 months of age. The gradual maturation of the myelin transcriptome up to the age of 6 months is also indicated by plotting the transcripts that are of particular abundance at any two (Fig. 4K and Fig. S4G) or three (Fig. 4L and Fig. S4G) of the analyzed ages.

To analyze how similar the top 1000 most abundant transcripts in myelin at any of the analyzed ages are to those in total brain regions we compared these transcriptomes and found about half of them (n = 555) also highly abundant in both cortex and cerebellum (Fig. 4M). Conversely, 548 transcripts are specifically enriched in myelin. Indeed, overall abundance in myelin is manifold higher compared to the homogenates of the analyzed brain regions (Fig. S4H).

### Stage specific expression kinetics suggest functional remodeling of the myelin transcriptome during development

Considering that the myelin transcriptome matures in mRNA composition and abundance during postnatal brain development we were curious whether this remodeling would indicate changes of the functional relevance of the mRNA-pool. We thus performed GO-term enrichment analysis for those transcripts that display a stage-specifically differential abundance (compare k-means clusters in Fig. 5A). Indeed, the clusters correspond to distinct biological processes (Fig. 5B–E). Transcripts most abundant in myelin at P18 are preferentially associated with biological processes involved in energy metabolism and nervous system development (Fig. 5B). Transcripts highly abundant in myelin at P75 display a strong association with protein translation, protein targeting, and RNA catabolism (Fig. 5C). The transcripts abundant in mature myelin at 6 and 24 months are preferentially associated with ion homeostasis and cellular signaling (Fig. 5D,E). The comparatively fewer transcripts most abundant in myelin at 6 months of age (n = 176) are also associated with vesicle-mediated transport and ribonucleotide metabolic processes (Fig. 5E).

Finally, we compared the myelin transcriptome with 77 genes causing (when mutated) brain disorders preferentially affecting the white matter (leukodystrophies/leukoencephalopathies). Surprisingly, most of the respective mRNAs were found in myelin at least at moderate abundance (Fig. 5F) (Supplementary Table 13); indeed, the majority of them were enriched in myelin compared to other tissues (Fig. S5).

Taken together, we report the existence of an unexpectedly large mRNA-pool in CNS myelin, which displays a maturation-dependent dynamic modulation with respect to composition, abundance, and functional associations.

## Discussion

In this study we obtained the first RNA-Seq transcriptome database of myelin purified from wild-type mouse brains at various ages (P18, P75, 6mo and 24mo). We report that CNS myelin comprises an unexpectedly large number of mRNAs, with transcripts of >13000 mRNAs present at least at a moderate abundance level; a surprising finding when considering that only very few mRNAs have been previously recognized in myelin using a small-scale approach^6^. Indeed, >1000 transcripts were specifically enriched in myelin compared to other brain tissues. The selectivity of mRNA incorporation into myelin and thus the specificity of the myelin transcriptome is also indicated by our finding that myelin-enriched transcripts are not necessarily among those most abundantly expressed in oligodendrocytes when analyzed by comparing our dataset with the previously determined transcriptomes of various cell types of the brain^28^. *Vice versa*, mRNAs highly expressed in the oligodendrocyte lineage are not necessarily enriched in myelin. As expected, mRNA markers of neurons, astrocytes and microglia are diminished in purified myelin. Together this indicates that the identified mRNA-pool in the myelin compartment is unique and distinct from that in surrounding brain tissue and in oligodendroglial cell bodies.

Incorporation of transcripts into myelin is mediated by the microtubule-dependent transport of RNA-granules that contain ribosomes, regulatory proteins, and a selection of mRNAs^9^,^29^. Considering that MBP is rate-limiting for myelination^11^,^12^, the molecular control of *Mbp*-mRNA translation appears crucial to regulate the precise extent of myelin biogenesis. Indeed, *Mbp*-mRNA is transported into myelin sheaths along microtubules^19^,^20^,^30^. Its translation is confined to MSAS by translation-inhibitory factors, including ribonucleoproteins^21^,^22^,^23^ and the small non-coding RNA 715^31^. Considering that MBP is the second-most abundant structural myelin protein^32^, it is not surprising that *Mbp*-mRNA is strongly enriched in myelin. We note that *Mobp*-mRNA, which is also highly enriched in myelin, is also recognized by trans-acting ribonucleoproteins and transported in RNA-trafficking granules along microtubules^33^. Excessive microtubule accumulation in oligodendrocytes in the hypomyelinating *taiep* mutant rat^34^, causes impaired RNA-granule dynamics^35^ and the accumulation of both, *Mbp* and *Mobp* mRNAs in oligodendroglial cell bodies^36^. Together, the mechanisms underlying the incorporation of transcripts into myelin have largely been established using *Mbp*-mRNA but probably apply to other myelin-related transcripts as well. This has most recently been exemplified for the mRNA encoding the small heat-shock protein α-B-crystallin (CRYAB, also termed HspB5). The rapidly increased abundance of CRYAB upon a neuroinflammatory challenge protects myelin and neurons from degeneration^37^. By *in situ*-hybridization, *Cryab*-mRNA in oligodendrocytes localized away from the cell bodies^38^, in resemblance of *Mbp*-mRNA^24^. Indeed, *Cryab*-mRNA is enriched in myelin according to the present analysis. Its continuous availability for rapid local protein translation in the individual internode may be advantageous upon an immunological challenge.

Myelin remodeling is now recognized as a mechanism of life-long brain plasticity^39^ with consequences for the velocity of nerve conduction and thus cognitive and motor capabilities. Indeed, it has long been known that neuronal activity can stimulate central myelination^5^,^40^. It is hypothesized that the *en passent* release of glutamatergic vesicles from active axons towards adjacent cells of the oligodendrocyte lineage^41^,^42^ enhances the synthesis of MBP locally^43^,^44^, i.e. in the individual internode. Apparently, thus, electrically active axons have an advantage over neighboring silent axons in the induction of active myelination by their associated oligodendroglial processes. Considering that individual oligodendrocytes commonly myelinate segments of numerous axons, the local control of myelination at the level of the individual internode appears well suited to modulate the local extent of myelination in dependence of axonal activity.

The locally controlled translation of myelin-enriched *Mbp*-mRNA facilitates the MBP-dependent compaction of newly synthesized myelin membrane at MSAS distant from the oligodendroglial cell body^45^. Indeed, the existence of many additional myelin-enriched mRNAs (as reported here) may thus indicate the existence of a reservoir for the local translation of further myelin proteins for the turnover or adaptive modulation of myelin. More speculatively, the substantial overlap between the myelin transcriptome and the myelin proteome may reflect that most myelin constituents can be biosynthesized locally at MSAS. Interestingly, beyond myelin-related mRNAs, mRNAs encoding proteins required for protein translation and trafficking are also enriched in myelin. This may enable myelin to extend its own protein translation machinery without an immediate requirement for the comparatively slower intracellular transport^7^ from the cell body through the oligodendroglial processes to the MSAS in non-compact myelin. It is interesting to note that the mRNA-pool of CNS myelin displays considerable changes in composition and abundance during myelin maturation whereas ageing-dependent changes at over six months of age were minor.

The present RNA-Seq dataset provides a valuable reference for investigating oligodendroglial mRNA transport mechanisms, as well as myelin maturation, turnover, adaptive modulation, and ageing. Considering the robustness and reproducibility of both, biochemical myelin purification and RNA-Seq, the methods will be useful for applications beyond wild-type myelin. For example, myelin can be biochemically purified from mutant mice^32^ that model neurological disorders. In recent years it has become increasingly evident that myelin and myelinating cells are pathophysiologically involved in many neurological disorders, including multiple sclerosis, leukodystrophies, Rett syndrome, amyotrophic lateral sclerosis, and psychiatric diseases^25^,^46^,^47^,^48^,^49^,^50^. We believe that the combination of myelin purification with unbiased transcriptome and proteome analysis can provide molecular profiles that hold great promise for better understanding their pathomechanisms.

## Exeprimental Procedure

### Biochemical analysis

A light-weight membrane fraction enriched for myelin was purified from mouse brains as described^51^. Male c57Bl6/N mice from the breeding colony of the Max Planck Institute of Experimental Medicine (MPIEM, Göttingen, Germany) were used. For immunoblot, mice were 75 days old. For proteome analysis and qRT-PCR, mice were 6 months old. For RNA-Seq, indicated ages were used. Animal experiments were performed in accordance with the animal policies of the Max Planck Institute of Experimental Medicine (Göttingen, Germany) approved by the institute's animal welfare officer and the Landesamt für Verbraucherschutz und Lebensmittelsicherheit (LAVES), the responsible authority for the German Federal State of Niedersachsen. The protein concentration was determined using the DC protein assay (BioRad). Immunoblotting was as described^52^. Antibodies were specific for PLP/DM20 (A431^53^, 1:5000), MBP (Dako A0623, 1:500), CNP (Sigma C 5922, 1:1000), SIRT2 (Abcam ab67299, 1:500), OLIG2 (DF308^54^, 1:200, kindly provided by J. Alberta and C. Stiles, Boston, MA, USA) GFAP (Novocastra NCL-GFAP-GA5, 1:500), AIF (also termed IBA1; Abcam ab107159, 1:500), GPM6A (#24924^55^, 1:1000), TUBB3 (also termed TUJ1; Covance MMS-435P, 1:1000), and VDAC (Rockland 600-401-882, 1:2000). Immunoblots were scanned using the Intas ChemoCam system.

### qRT-PCR

RNA was extracted from brain lysates and purified myelin using the miRNeasy Mini Kit (Qiagen). cDNA was synthesized using the Transcriptor High Fidelity Reverse Transcription Kit and Random Hexameric Primers (Roche Applied Science). qRT-PCR was performed using the TaqMan Fast Advanced Master Mix according to manufacturer’s protocol in a 96-well plate StepOnePlus Real-Time PCR System (both Applied Biosystems). Primers and Universal Probe Library (UPL) probes were specific for *Mbp* (forward 5′-AACATTGTGA CACCTCGAACA, reverse 5′-TGTCTCTTCC TCCCCAGCT, UPL probe #58), *Mobp* (forward 5′-GGCTCTCCAA GAACCAGAAG, reverse 5′-GCTTGGAGTT GAGGAAGGTG, UPL probe #74), *Fth1* (forward 5′-TGGAGTTGTA TGCCTCCTACG, reverse 5′-TGGAGAAAGT ATTTGGCAAAGTT, UPL probe #21), *Itgam* (forward 5′-GGAGCCCCAC ACTAGCATCAA, reverse 5′-CAAAGGGAGG CCCCAAAATAAG), *Tgfb1* (forward 5′-CAAGTGTGGA GCAACATGTGGAA, reverse 5′-CGTATCAGTG GGGGTCAGCAG), *Tlr7* (forward 5′-GGATGATCCT GGCCTATCTCTGA, reverse 5′-TCCGTGTCCA CATCGAAAACAC), *L1cam* (forward 5′-CAGCCTGCCT TCAGACCATCA, reverse 5′-ATGTTCTGGG GATTCTTGTCTGG), *Syt1* (forward 5′-GCGATCTCCA GAGTGCTGAGAAA, reverse 5′-ACAGTCAGCT TGCCGGCAGTA), *Kcna4* (forward 5′-GAAGGCACTG GGGGTTCTGGT, reverse 5′-AGTAGGCCCC ACGTGTCTGATG), *Slc4a4* (forward 5′-CACGAAGAAC GCCAGGAC, reverse 5′-TCCGGTACTT CCTGTGGAAC), *Itga7* (forward 5′-GTCCGTGCTC TGGACTCTGTGG, reverse 5′-CCCAGCTCAC ACTCGACATGA), and *Hgf* (forward 5′-CACTGACCCA AACATCCGAGTTG, reverse 5′-TCCCATTGCCACGATAACAATCT).

### RNA-Seq data analysis

RNA-Seq data was generated using Illumina sequencing. Reads were aligned to mouse genome (mm9) using TopHat^56^ (version 2.0.9) with default options. The aligned reads were then provided as an input for the HTSeq_count utility from the HTSeq package. The raw read count files obtained from HTSeq-count were then processed for differential expression using the DESeq package^57^. Expression was quantified after library size normalization using DESeq^57^. Differential expression analysis was performed using the DESeq package with a FDR rate of 0.1^57^.

### Accession numbers

The next generation sequencing datasets generated in this study have already been submitted to GEO and will be publically available under accession number GSE78151. Tissue-specific datasets for cerebellum, cortex, heart, kidney, liver and lung were obtained from the ENCODE project deposited in Gene Expression Omnibus (GEO) with accession GSE36026. All these data sets were processed as described above.

### Myelin proteome analysis

Myelin purified from the brains of 6 months old mice (see above) was homogenized in RIPA buffer and samples were boiled at 70 °C for 10 minutes in 1x NuPAGE LDS Sample Buffer (Life technologies) supplemented with 100 mM DTT. The samples were separated on a 10% NuPAGE Bis-Tris gel (Life technologies) for 20 minutes at 180V in MOPS running buffer (Life technologies), fixated in 7% acetic acid containing 40% methanol and subsequently stained for 30 minutes using Colloidal Blue staining kit (Life technologies) and excised from the gel. Protein lane was divided into four slices. Each slice was chopped and destained (50% ethanol in 25 mM NH_4_HCO_3_) for 15 minutes rotating at room temperature and dehydrated for 10 minutes rotating in 100% acetonitrile. Vacuum dried samples were rehydrated and reduced for 60 minutes in reduction buffer (10 mM DTT in 50 mM NH_4_HCO_3_ pH 8.0) at 56 °C and subsequently alkylated in 50 mM iodoacetamide in 50 mM NH_4_HCO_3_ pH 8.0 for 45 minutes at room temperature in the dark. Dehydrated (10 minutes rotating in 100% acetonitrile) and vacuum dried samples were subjected to trypsin digestion (1 μg trypsin/sample in 50 mM NH_4_HCO_3_ pH 8.0) at 37 °C over night. Peptides were extracted twice in extraction solution (30% acetonitrile) and once in 100% acetonitrile for 15 minutes at 25 °C shaking at 1400 rpm, purified and desalted using C18 stage tips. Peptides were separated on C18 columns (New Objective) with inner diameter of 75 μm packed with 1.9 μm Reprosil beads (Dr. Maisch) that was mounted to an EasyLC1000 HPLC (Thermo). Eluting peptides (Buffer A: 0.1% formic acid, Buffer B: 80% acetonitrile and 0.1% formic acid, Gradient: 0–67 min 0–22% buffer B, 68–89 min 22–40% buffer B, 90–95 min 40–95% buffer B) were directly sprayed into a Q Exactive Plus mass spectrometer from Thermo operating in positive scan mode with a full scan resolution of 70,000; AGC target 3 × 10^6; max IT = 20ms; Scan range 300–1650 m/z and a Top10 MSMS method. Database search was performed using MaxQuant Version 1.5.2.8^58^ against Mouse Uniprot database downloaded on 8. January 2015 (75,403 entries), with Trypsin/P as digestion enzyme allowing 2 missed cleavages. As settings the following was applied: variable modification: Acetyl (Protein N-term);Oxidation (M), fixed modifications: Carbamidomethyl (C), FDR of 1% on peptide and protein level was applied. Proteins with at least two peptides (one of them unique) were considered as identified. Proteins matching reverse database or common contamination list as well as proteins with peptides only identified by peptides with modification were filtered out.

## Additional Information

**How to cite this article**: Thakurela, S. *et al*. The transcriptome of mouse central nervous system myelin. *Sci. Rep.* **6**, 25828; doi: 10.1038/srep25828 (2016).

## Supplementary Material

Supplementary Information
