# Supplementary material for: The transcriptome of mouse central nervous system myelin
Source: Sci Rep. 2016 May 13;6:25828. doi: 10.1038/srep25828 (PMC4865983; doi:10.1038/srep25828)

The transcriptome of mouse central nervous system myelin

Sudhir Thakurela[1#], Angela Garding[1#], Ramona B. Jung[2], Christina Müller[3], Sandra Goebbels[2], Robin White[3], Hauke B. Werner[2,*], Vijay K. Tiwari[1,*]

1. Institute of Molecular Biology (IMB), 55128 Mainz, Germany
2. Department of Neurogenetics, Max Planck Institute of Experimental Medicine, 37075 Göttingen, Germany
3. Institute of Physiology, University Medical Center of the Johannes Gutenberg-University, 55128 Mainz, Germany

[#] equal first authors

* Shared senior authorship. Correspondence should be addressed to hauke@em.mpg.de or v.tiwari@imb-mainz.de

LEGENDS FOR SUPPLEMENTARY FIGURES

Supplementary Fig 1:

A. Matrix scatter plot showing Spearman correlation between biological replicate samples of myelin (age 6 months).

B. Matrix scatter plot showing Pearson correlation between biological replicate samples of myelin (6 months).

C-N. UCSC browser tracks showing mRNA reads in relation to the *Cnp, Mag, Sirt2, Car2, Cryab, Mobp, Mog, Padi2 and Plp1* genes.

Supplementary Fig 2:

Heat map showing the abundance of the top 30 myellin transcripts in myelin (6 months), cerebellum, cortex, heart, kidney, liver, and lung.

Supplementary Fig 3:

A. Dendrogram comparing the transcriptome of myelin with that of cerebellum and cortex.

B. Venn diagram of transcripts that are at least 2-fold more abundant in myelin compared to cerebellum, cortex, or both.

C. Heat map of mRNAs that are at least 2-fold more abundant in myelin compared to both cerebellum and cortex.

D. Heat map of transcripts that are at least 2-fold more abundant in myelin compared to either cerebellum or cortex.

E. Transcripts detected in oligodendrocyte progenitor cells (OPC), newly formed oligodendrocytes (NFO) and myelinating oligodendrocytes (MO) according to Zhang et al (2014) were displayed in a density plot to categorize mRNA abundance levels (x-axis) in relation to the

frequency of occurrence (y-axis). Vertical lines denote FPKM cutoffs to categorize transcripts as not expressed (NE), lowly expressed (Low), moderately expressed (Mod), or highly expressed (High).

F. Box-Whisker plot comparing the abundance of transcripts in myelin (age 6 months) with that of the top 1000 most abundant mRNAs in OPC, NFO and MO.

G. Combined ranked, clustered heat map of the top 500 most abundant mRNAs expressed in myelin 6 month, astrocytes, neurons and microglia[1]. The transcripts with highest abundance in myelin do not display highest abundance in the majority of surrounding cell types.

H. Bar plot showing the enrichment of biological processes for those genes of which the products were identified in myelin at both, the mRNA-level (by RNA-Seq) and the protein level (by mass spectrometry). Bars indicate number of genes in a category (lower x-axis) while the line represents p-value for each biological term (upper x-axis).

I. Venn diagram comparing all transcripts detected by RNA-Seq with all proteins identified by mass spectrometry in myelin (6 months).

Supplementary Fig 4:

A. Bar plot showing enrichment of biological processes in transcripts that are among the top 1000 most abundant in myelin at any age.

B. Bar plot showing enrichment of pathways in transcripts that are among the top 1000 most abundant in myelin at any age.

C. Bar plot showing enrichment of known mouse mutant phenotypes in genes that are among the top 1000 most abundant in myelin at any age.

D. Bar plot showing enrichment of biological processes in transcripts that are significantly increased in abundance by at least 2-fold between P18 and P75 and are also detected above the normalized read count of 100.

E. Bar plot showing enrichment of biological processes in transcripts that are significantly increased in abundance by at least 2-fold between P75 and 6 months and are also detected above normalized read count.

F. Bar plot showing enrichment of biological processes in transcripts that are significantly increased in abundance by at least 2-fold between 6 and 24 months of age and are also detected above normalized read count.

G. Box-Whisker plots comparing transcript abundance for genes that are among the top 1000 most abundant in myelin at any one, two or three analyzed ages.

H. Box-Whisker plot comparing the top 1000 most abundant transcripts in myelin at any of the analyzed ages with that in total cerebellum and cortex.

Supplementary Fig 5:

Heat map displaying the abundance in myelin and other tissues of mRNAs associated with leukodystrophies / leukoencephalopathies. Note that the majority of analyzed transcripts are enriched in myelin compared to other tissues.

LEGENDS FOR SUPPLEMENTARY TABLES

Supplementary table 1. Table providing abundance of transcripts in the four stages of myelin identified by RNA-Seq.

Supplementary table 2. Gene up-regulated in myelin as compared to cortex.

Supplementary table 3. Gene up-regulated in myelin as compared to cerebellum.

Supplementary table 4. Highly expressed (top 1000) myelin genes and their expression in different stages of oligodendrocytes.

Supplementary table 5. Highly expressed genes in myelinating oligodendrocytes and their expression in myelin.

Supplementary table 6. Proteins identified in 6 month myelin by mass-spectroscopy.

Supplementary table 7. List of genes overlapping in transcriptome and proteome.

Supplementary table 8. Genes differentially expressed with at least two fold change and expression above 100 normalized read count between P18 and P75 ages.

Supplementary table 9. Genes differentially expressed with at least two fold change and expression above 100 normalized read count between P75 and 6 months.

Supplementary table 10. Genes differentially expressed with at least two fold change and expression above 100 normalized read count between 6 months and 24 months.

Supplementary table 11. Table providing genes from individual k-means clusters from Fig. S3G.

Supplementary table 12. List of top 1000 genes at 6 months of myelin.

Supplementary table 13. List of overlapping genes between genes known for leukodystrophies / leukoencephalopathies and genes found to be abundant in 6 months myelin.

1. Zhang, Y. et al. An RNA-sequencing transcriptome and splicing database of glia, neurons, and vascular cells of the cerebral cortex. *J Neurosci* **34**, 11929-11947 (2014).

Supplementary Figure 1

Thakurela et al

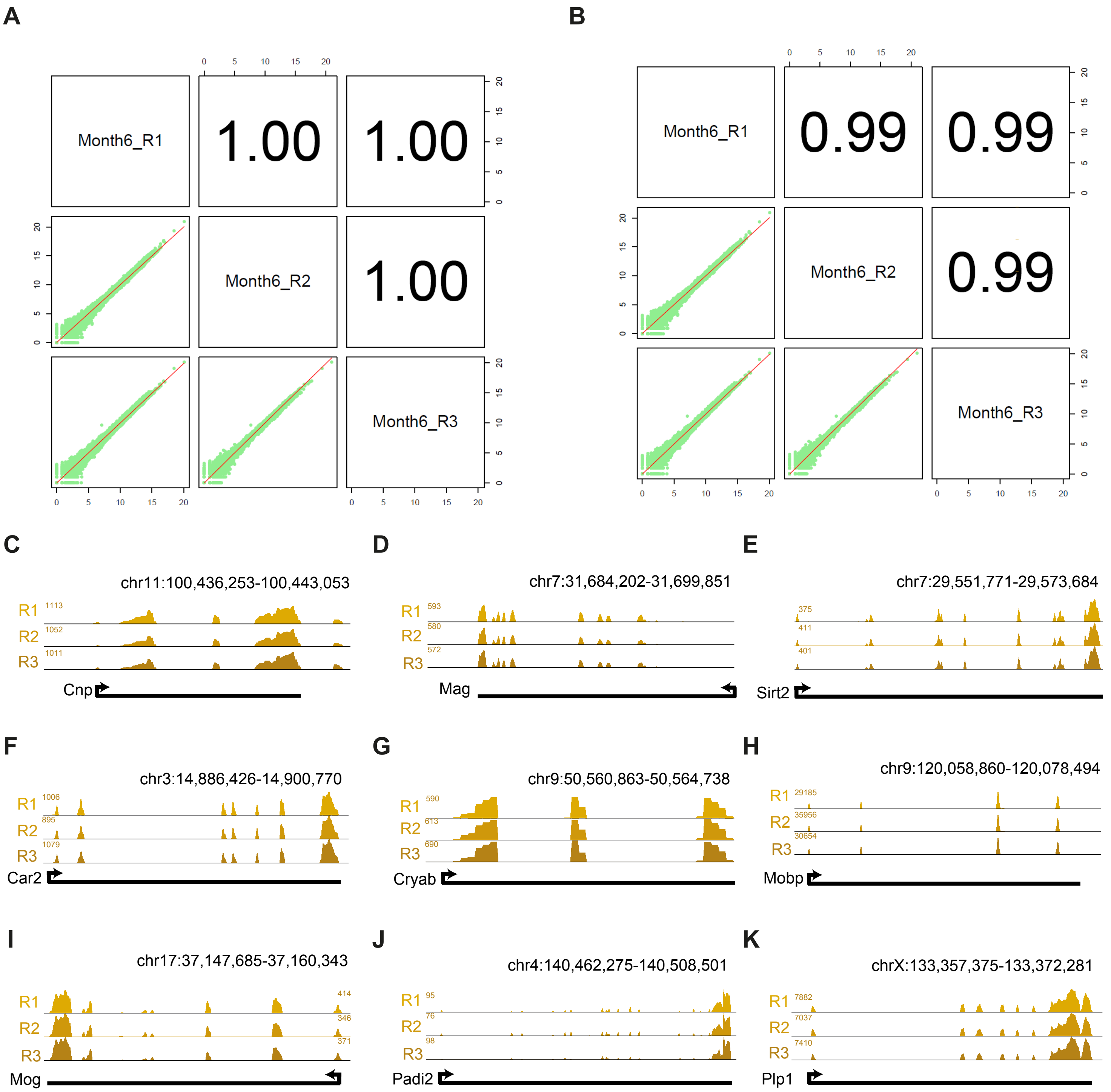

Supplementary Figure 2

Thakurela et al.

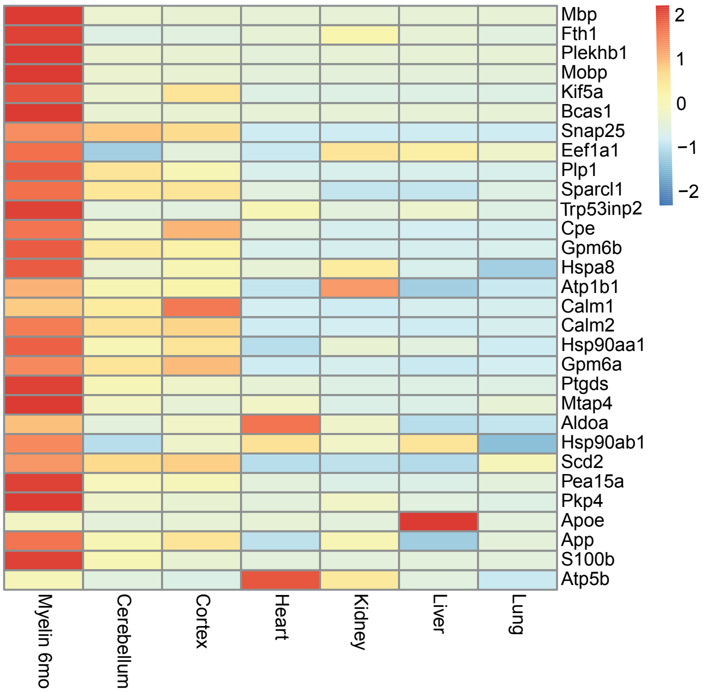

Supplementary Figure 3

Thakurela *et. al.*

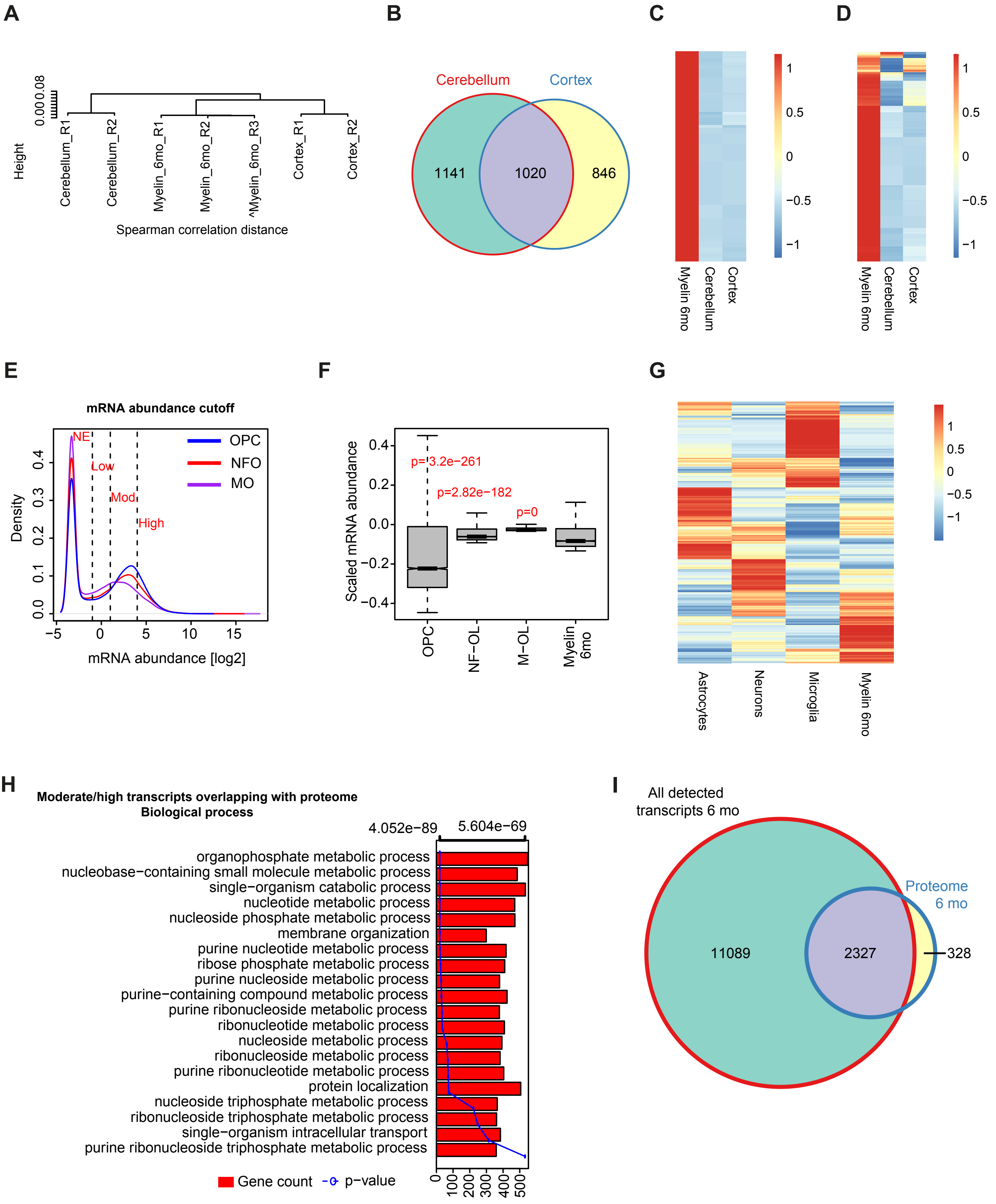

Supplementary Figure 4

Thakurela et al

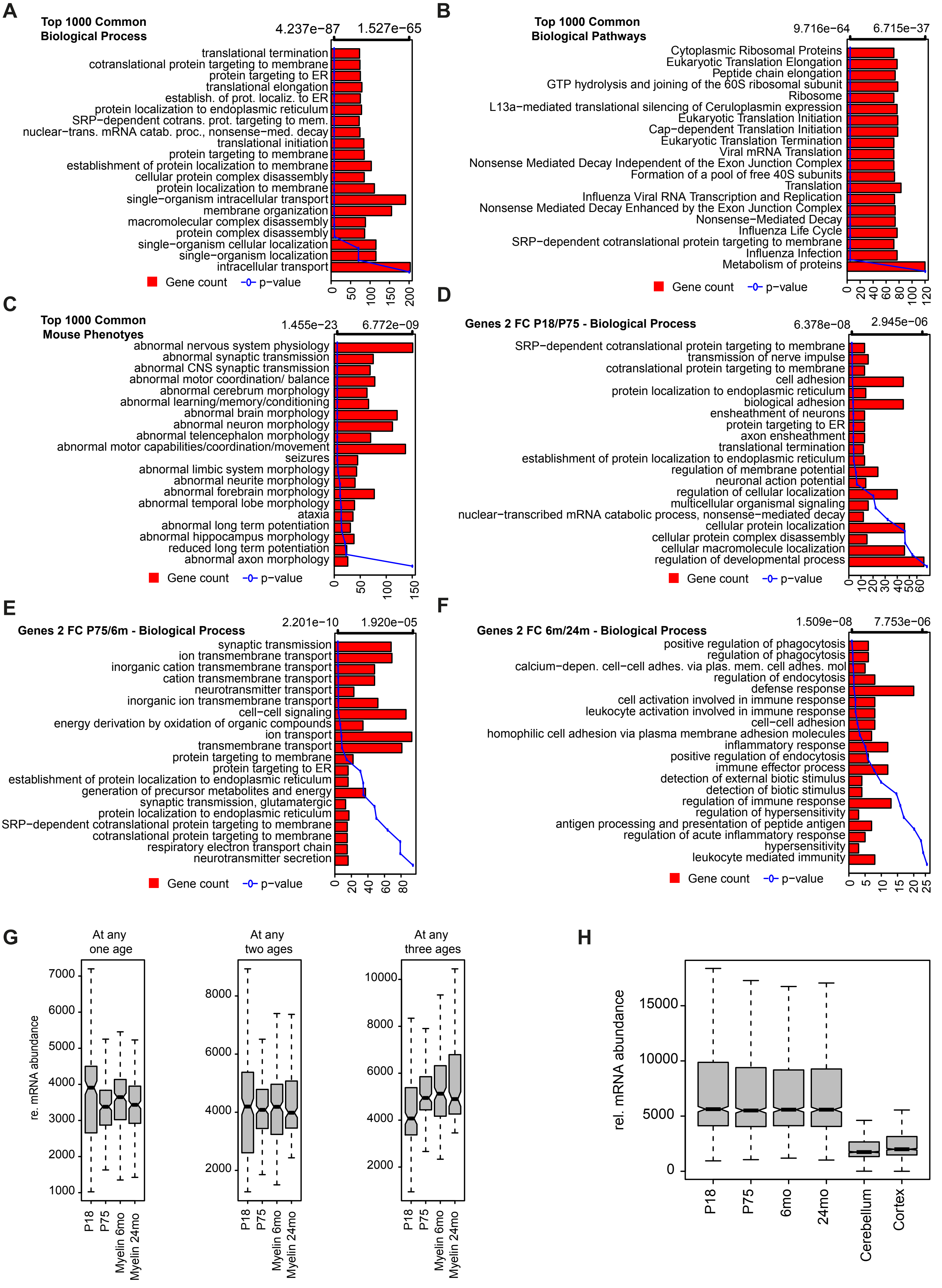

Supplementary Figure 5 **Thakurela et al**

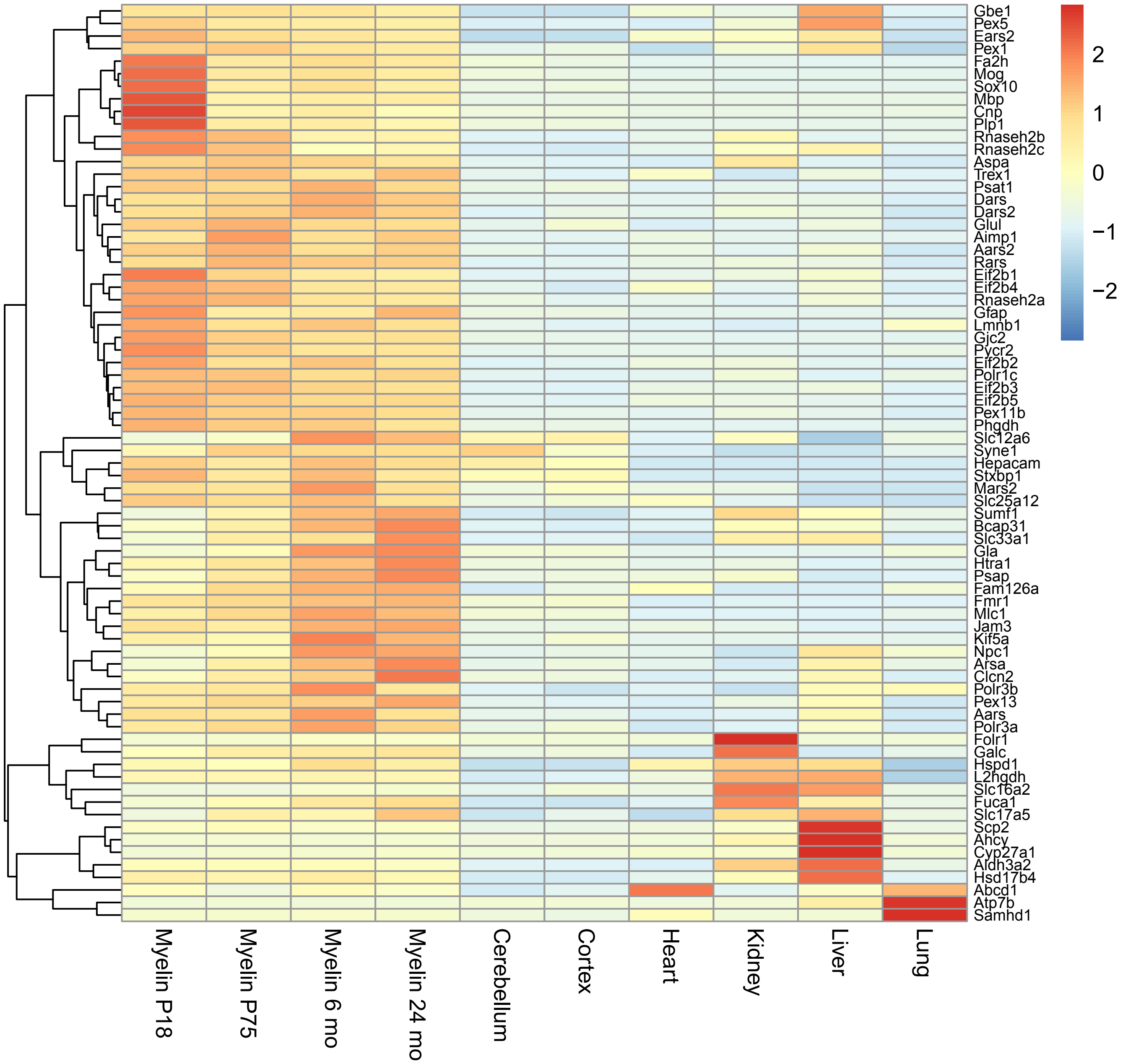

Supplement: Supplementary Information [file srep25828-s1.pdf]
